# Genetic association studies and the effect of misclassification and selection bias in putative confounders

**DOI:** 10.1186/1753-6561-3-s7-s48

**Published:** 2009-12-15

**Authors:** Christy L Avery, Keri L Monda, Kari E North

**Affiliations:** 1 Department of Epidemiology, University of North Carolina, 137 East Franklin Street, CB #8050, Suite 306, Chapel Hill, North Carolina 27514 USA; 2 Carolina Center for Genome Sciences, University of North Carolina, 5009 Genetic Medicine Building, Chapel Hill, North Carolina 27599 USA

## Abstract

Genetic epidemiology studies often adjust for numerous potential confounders, yet the influences of confounder misclassification and selection bias are rarely considered. We used simulated data to evaluate the effect of confounder misclassification and selection bias in a case-control study of incident myocardial infarction. We show that putative confounders traditionally included in genetic association studies do not alter effect estimates, even when excessive levels of misclassification are incorporated. Conversely, selection bias resulting from covariates affected by the single-nucleotide polymorphism of interest can bias effect estimates upward or downward. These results support careful consideration of how well a study population represents the target population because selection bias may result even when associations are modest.

## Background

Genetic epidemiology studies often adjust for numerous covariates when estimating associations between genetic variants and an outcome of interest [1-3]. However, several authors have questioned whether acquired risk factors can confound genetic associations [4-6]. Others have suggested that confounders only influence genetic associations through selection bias [7,8]. We therefore initially investigated whether acquired risk factors confounded the association between two simulated causal single-nucleotide polymorphisms (SNPs) and the odds of myocardial infarction (MI) using the Genetic Analysis Workshop 16 simulated data. This platform facilitated an evaluation of non-differential and differential misclassification in potential confounders, as well as selection bias.

## Methods

### Study population and sources of data

The Framingham Heart Study simulated dataset provided included 6,476 Caucasians from 1,129 pedigrees. Individuals were genotyped using a 50 k gene-focused panel and a 500 k Human Mapping Array Set provided by Affymetrix. We selected unrelated participants from the cohort and offspring populations (*n *= 1,741) and examined two SNPs using a dominant genetic model: rs12565497, simulated association with MI (Scenario 1, the investigation of differential and non-differential misclassification), and rs1466535, simulated association with low-density lipoprotein (LDL) concentrations (Scenario 2, the investigation of selection bias). MI was defined as any MI over the three simulated study exams. Baseline LDL concentrations dichotomized at 160 mg/dl were used to examine the effect of selection bias. Baseline smoking history and medication use (Rx) were used to evaluate misclassification.

### Statistical methods

Confounders of the association between the SNP and MI were identified using two methods. First, we employed a directed acyclic graph (DAG), which allowed us to apply a simple set of rules to graphically encode relationships between study variables [9]. DAGs are particularly informative because they make untested assumptions between variables explicit so that their implications can be studied further. Second, we evaluated potential confounders using established criteria that required an association between the potential confounder and the exposure (i.e., the SNP) among those without MI as well as an association between the potential confounder and MI within the whole population [10]. Statistical tests were not used to identify confounding because they only evaluate the confounder-outcome association.

Non-differential misclassification in confounder measurement (i.e., confounder misclassification unrelated to disease) was examined several ways. First, we evaluated smoking history and Rx separately by including each variable in the initial model that only included rs12565497 and randomly changing the true covariate values in 0.5-25% of the population. We also evaluated the potential for joint non-differential misclassification by including both smoking and Rx in the initial model and independently altering 5-25% of the true covariate values for each putative confounder. To evaluate the effect of misclassification related to the true confounder values, we also considered models that fixed the probability of smoking misclassification for non-smokers and Rx (regardless of status) at 2% and varied the misclassification probability for smokers from 2-30%. We did not vary Rx misclassification by usage because we did not believe that the misreporting proportion would vary by usage status, unlike smoking. The low prevalence of usage (3.3%) also limited our analytic options.

Differential misclassification in confounder measurement (i.e., confounder misclassification related to disease) was also evaluated for each potential confounder separately and jointly. For example, differential misclassification in smoking history was examined by fixing the misclassification proportion for participants who did not smoke and did not develop a MI at 0.98. The misclassification proportion for persons who smoked and had a MI was varied from 0.70 to 0.85. Non-smokers with a MI or smokers without a MI were assigned intermediate probabilities of misclassification.

Selection bias (e.g., inappropriate control selection, healthy-worker bias, or non-response bias [11]) was investigated using rs1466535, simulated to influence LDL concentrations. First we estimated the rs1466535-MI relationship. We then introduced selection bias by randomly removing 5-60% of the following four populations: high LDL, low LDL, smokers, and non-smokers. We also evaluated the effect of selection bias related to smoking and LDL jointly by fixing the participation proportions for the low LDL population at 1.0 and the non-smoking population with high LDL at 0.8. The participation proportion for persons who smoked and had high LDL was then varied from 0.4-0.7. All analyses were performed unblinded to the answers using logistic regression, 500 iterations on the first simulated dataset in SAS 9 (Cary, NC).

## Results

The minor allele frequencies of rs12565497 and rs1466535 are 0.30 and 0.32, respectively. Both SNPs are in Hardy-Weinberg equilibrium. The estimated prevalences of MI, smoking, high LDL, and Rx are 26.1%, 24.3%, 67%, and 3.3%, respectively.

**Figure 1:**
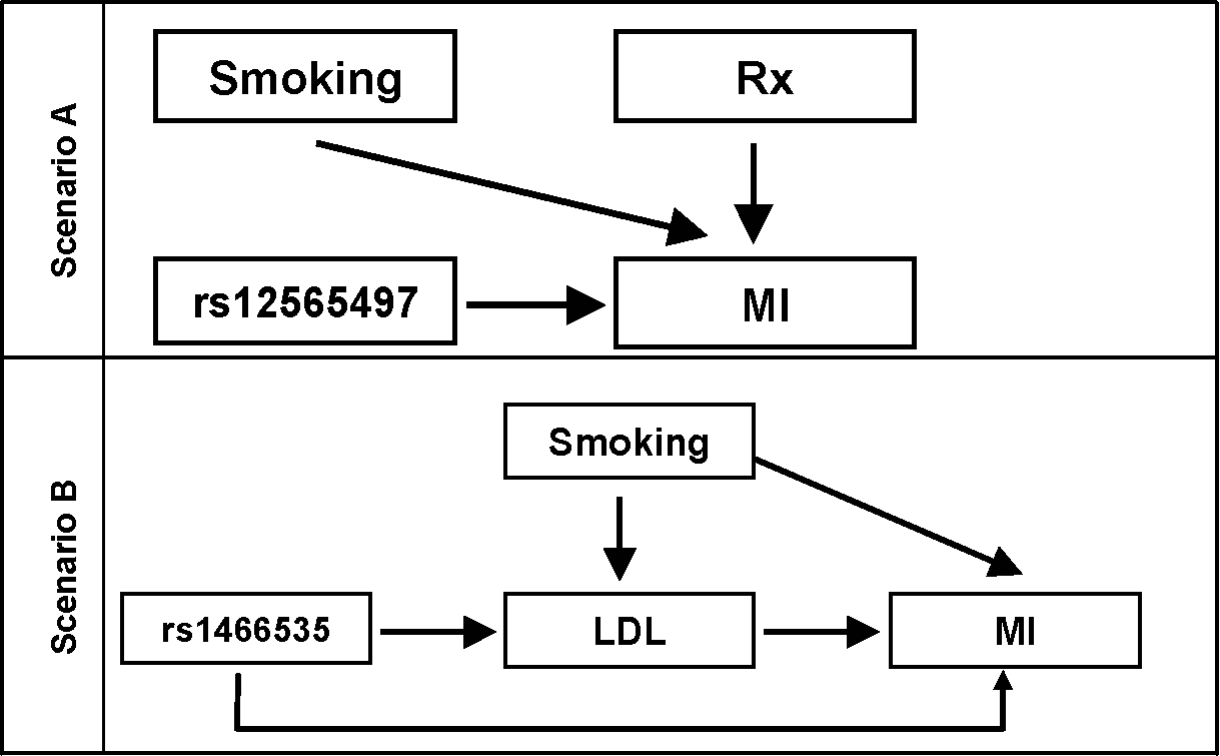
**DAGs detailing potential confounding (A) and selection bias (B) in a simulated case-control study of MI**.

Table 1 and Figure 1 present the relationships between the genetic variants, putative confounders, and MI from Scenarios A (confounder misclassification) and B (selection bias). The Scenario A DAG demonstrates that neither smoking nor Rx confounds the association between rs12555497 and MI because neither is associated with the SNP. This is supported by the odds ratios (OR) for rs12565497-smoking (OR = 0.95; 95% CI = 0.73, 1.25) and rs12565497-Rx (OR = 0.60; 95% CI = 0.25, 1.40). The estimates of the SNP-MI association adjusted for Rx (OR = 1.46; 95% CI = 1.17, 1.81), smoking (OR = 1.47; 95% CI = 1.19, 1.83), and Rx and smoking together (OR = 1.47; 95% CI = 1.18, 1.83) are also very similar to the unadjusted estimate (OR = 1.47; 95% CI = 1.18, 1.82), further suggesting that neither confounds the rs12555497-MI association (results not shown).

**Table 1:** Relationship between genetic variants, putative confounders, and MI used to examine misclassification (Scenario A) and selection bias (Scenario B)

Exposure	Outcome	Population	Estimate (95% CI)
Scenario A			
rs12565497	Smoking	MI = 0	0.95 (0.73, 1.25)
Smoking	MI	Total population	2.08 (1.48, 2.94)
rs12565497	Rx	MI = 0	0.60 (0.25, 1.40)
Rx	MI	Total population	2.62 (1.18, 5.80)
Scenario B			
rs1466535	Smoking	MI = 0	1.15 (0.88, 1.51)
Smoking	MI	rs1466535 AA^a^	2.03 (1.60, 2.57)
Smoking	LDL^b^	Total population	1.31 (1.03, 1.66)
rs1466535	LDL^b^	Total population	0.81 (0.66, 0.99)
rs1466535	Smoking	Total population	1.14 (0.91, 1.42)
rs1466535	MI	Total population	0.80 (0.64, 1.00)

^a^Referent group

^b^LDL dichotomized at 160 mg/dl

Due to space restrictions, only selected results of iterative analyses evaluating the effect of non-differential misclassification by jointly including smoking status and Rx are reported in Table 2. Results show that the estimate of the "truth" was included in the range generated by the 500 iterations, and that there was no bias in the SNP-MI estimate or standard error. We note that as the smoking misclassification proportion increases for smokers, the percent of estimates biased toward the null (defined as the proportion of estimates that were reduced in magnitude) increased.

**Table 2:** Effect of non-differential misclassification of smoking status and Rx on the rs12565497-MI association

% Misclassified			Average bias			
			
Smokers	Non-smokers	Rx	Estimate	SE	Range	% Biased toward null
**"Truth" (0% misclassification)**			**0.389**^a^	**0.111**^a^	**-**	**-**
2	2	2	0.003	0.001	0.377-0.407	25.8
5	2	2	0.003	0.001	0.371-0.413	27.2
10	2	2	0.002	0.001	0.372-0.411	34.8
20	2	2	0.002	0.001	0.361-0.417	42.4
30	2	2	0.001	0.000	0.363-0.421	45.0

^a^Denotes "true" estimate and standard error

As above, only selected results from differential misclassification analyses in which the probability of misreporting depended on both smoking status and the outcome are reported in Table 3. Iterative analyses suggested that differential misclassification does not bias the estimate of the SNP-MI association. In contrast to non-differential results, however, we note that as misclassification increases, the percent of estimates biased toward the null decreases. Results of other non-differential and differential misclassification analyses were consistent with these conclusions.

**Table 3:** Effect of differential misclassification of smoking status on the rs12565497-MI association

% Misclassified				Average bias			
			
MI smokers	MI non-smokers	No MI smokers	No MI non-smokers	Estimate	SE	Range	% Biased toward null
**"Truth" (Assumes 0% misclassification)**				**0.3889**^a^	**0.1111**^a^	**-**	**-**
15	5	10	2	-0.002	0.000	0.367-0.408	65.0
20	10	15	2	-0.001	0.000	0.360-0.421	56.6
25	15	20	2	-0.001	0.001	0.349-0.447	51.4
30	20	25	2	0.000	0.002	0.332-0.451	51.2

^a^Denotes "true" estimate and standard error

The Scenario B DAG (Figure 1) suggests that smoking and LDL do not confound the rs1466535-MI association (not shown). While rs1466535 is associated with LDL (Table 1), LDL is on the causal pathway; adjusting for it could bias the rs1466535-MI association or induce confounding [12]. The DAG also illustrates the potential for selection bias related to LDL levels. For instance, if LDL levels influence participation, a non-random sample of the target population could bias estimates of the rs1466535-MI association. Results in Table 4 show that decreasing the participation proportion for the high LDL or non-smoking populations negatively biases the rs1466535-MI association; this bias is larger for high LDL. Conversely, positive bias is observed when decreasing either the participation proportion for the low LDL or smoking populations. Results of other selection bias analyses were consistent with these conclusions.

**Table 4:** Selection bias in the rs1466535-MI association caused by LDL or smoking

	Population assessed for selection bias (100% participation assumed for other subpopulations)			
	
% participating	High LDL	Low LDL	Smoker	Non-smoker
**Truth^a^**	**-0.219**	**-0.219**	**-0.219**	**-0.219**
95	**-**0.002	0.002	0.002	**-**0.002
90	**-**0.005	0.004	0.004	**-**0.004
80	**-**0.012	0.009	0.007	**-**0.007
70	**-**0.019	0.014	0.010	**-**0.008
60	**-**0.030	0.019	0.014	**-**0.013
50	**-**0.049	0.023	0.018	**-**0.019
40	**-**0.069	0.028	0.022	**-**0.023

^a^Model with 100% participation

## Discussion

In this paper, we evaluated how misclassification and selection bias affected putative confounders in genetic association studies. We demonstrated that putative confounders did not alter effect estimates. Conversely, selection bias resulting from covariates affected by the SNP of interest could bias effect estimates upward or downward, the degree of bias and its direction depending on underlying relationships between the SNP, intermediate variable, and the outcome of interest.

A true confounder is defined as a variable that affects both the exposure and the outcome [10]. In this simulation study we used both smoking status and Rx as putative confounders. These confounders did not meet our criteria of "true" confounders in that there was no association between the confounder and the SNP among those that did not have an MI, although there was an association between the confounder and MI (see Table 1). Similarly, smoking and Rx did not fit conventional criteria as established by the DAG (Scenario A, Figure 1). Nonetheless, such covariates are often included in statistical models; authors state that they are eliminating excess variation, following standards set by the literature, or provide other rationales. Given that the majority of variables are measured with some degree of error, which may lead to misclassification, we hypothesized that explicitly introducing misclassification would alter the SNP-MI association. However, we did not find this, even when misclassifying substantial proportions of the population. Other covariates may function as true confounders in this association, among them race or study center, and might introduce bias if measured with systematic error. Such variables were not available in these data.

Ideal referent groups in case-control studies are populations that approximate the population from which the cases arose (i.e., the target population) [13,14]. As such, the relevant comparison to assess the adequacy of the control group is not a contrast of the controls and the cases, but a contrast of the controls and the target population. Using a simulation study of MI, we show that the selection of controls on factors (LDL) affected by the exposure of interest (SNP) can bias estimates upward or downward. The degree of bias and its direction depended on underlying relationships between the SNP, LDL, and MI (Figure 1). On the basis of our results, we caution against the use of poorly characterized control groups for which the researcher has little information to assess how well participants represent the target population. For instance, the use of "healthy controls" in genetic association studies has been recently popularized in genome-wide association studies and it is unknown how well these control groups represent the underlying target populations. We believe this deserves greater consideration because bias was demonstrated even though the simulated association between the intermediate variable and outcome of interest was modest.

## Conclusion

Traditionally included confounders such as smoking and Rx did not alter effect estimates, even with widely varying levels of differential and non-differential misclassification. However, selecting participants based on covariates affected by the SNP of interest biased effect estimates in both directions. Our findings support the need for careful consideration of how well a study population represents a target population because bias resulted even though the simulated association between rs1466535 and LDL was modest.

## List of abbreviations used

DAG: Directed acyclic graph; LDL: Low-density lipoprotein; MI: Myocardial infarction; OR: Odds ratio; Rx: Medication use; SNP: Single-nucleotide polymorphism;

## Competing interests

The authors declare that they have no competing interests.

## Authors' contributions

CLA and KLM performed statistic analysis, interpreted results, and collaborated in writing the manuscript; KEN aided in interpretation of results and collaborated in writing the manuscript. All authors read and approved the final manuscript.
